# Latin American Natural Product Database (LANaPDB): An Update

**DOI:** 10.1021/acs.jcim.4c01560

**Published:** 2024-11-06

**Authors:** Alejandro Gómez-García, Daniel A Acuña Jiménez, William J Zamora, Haruna L Barazorda-Ccahuana, Miguel Á. Chávez-Fumagalli, Marilia Valli, Adriano D Andricopulo, Vanderlan da S Bolzani, Dionisio A Olmedo, Pablo N Solís, Marvin J Núñez, Johny R Rodríguez Pérez, Hoover A Valencia Sánchez, Héctor F Cortés Hernández, Oscar M Mosquera Martinez, José L Medina-Franco

**Affiliations:** † DIFACQUIM Research Group, Department of Pharmacy, School of Chemistry, Universidad Nacional Autónoma de México, Avenida Universidad 3000, Mexico City 04510, Mexico; ‡ CBio3 Laboratory, School of Chemistry, University of Costa Rica, San Pedro, San José 11501-2060, Costa Rica; § Laboratory of Computational Toxicology and Artificial Intelligence (LaToxCIA), Biological Testing Laboratory (LEBi), University of Costa Rica, San Pedro, San José 11501-2060, Costa Rica; ∥ Advanced Computing Lab (CNCA), National High Technology Center (CeNAT), Pavas, San José 1174-1200, Costa Rica; ⊥ Computational Biology and Chemistry Research Group, Vicerrectorado de Investigación, Universidad Católica de Santa María, Arequipa 04000, Peru; # School of Pharmaceutical Sciences of Ribeirao Preto (FCFRP), University of São Paulo (USP), Avenida Professor Doutor Zeferino Vaz, s/n, Ribeirao Preto 14040-903, SP, Brazil; ∇ Laboratory of Medicinal and Computational Chemistry (LQMC), Centre for Research and Innovation in Biodiversity and Drug Discovery (CIBFar), São Carlos Institute of Physics (IFSC), University of São Paulo (USP), Av. João Dagnone, 1100, São Carlos 13563-120, SP, Brazil; ○ Nuclei of Bioassays, Biosynthesis and Ecophysiology of Natural Products (NuBBE), Department of Organic Chemistry, Institute of Chemistry, São Paulo State University (UNESP), Av. Prof. Francisco Degni, 55, Araraquara 14800-900, SP, Brazil; ◆ Center for Pharmacognostic Research on Panamanian Flora (CIFLORPAN), College of Pharmacy, University of Panama, Av. Manuel E. Batista and Jose De Fabrega, Panama City 3366, Panama; ¶ Natural Product Research Laboratory, School of Chemistry and Pharmacy, University of El Salvador, Final Ave. Mártires Estudiantes del 30 de Julio, San Salvador 01101, El Salvador; ▶ GIFAMol Research Group, School of Chemistry Technology, Universidad Tecnológica de Pereira, Pereira 660003, Colombia; ▷ GIEPRONAL Research Group, School of Basic Sciences, Technology and Engineering, Universidad Nacional Abierta y a Distancia, Dosquebradas 661001, Colombia; ◀ GBPN Research Group, School of Chemistry Technology, Universidad Tecnológica de Pereira, Pereira 660003, Colombia

## Abstract

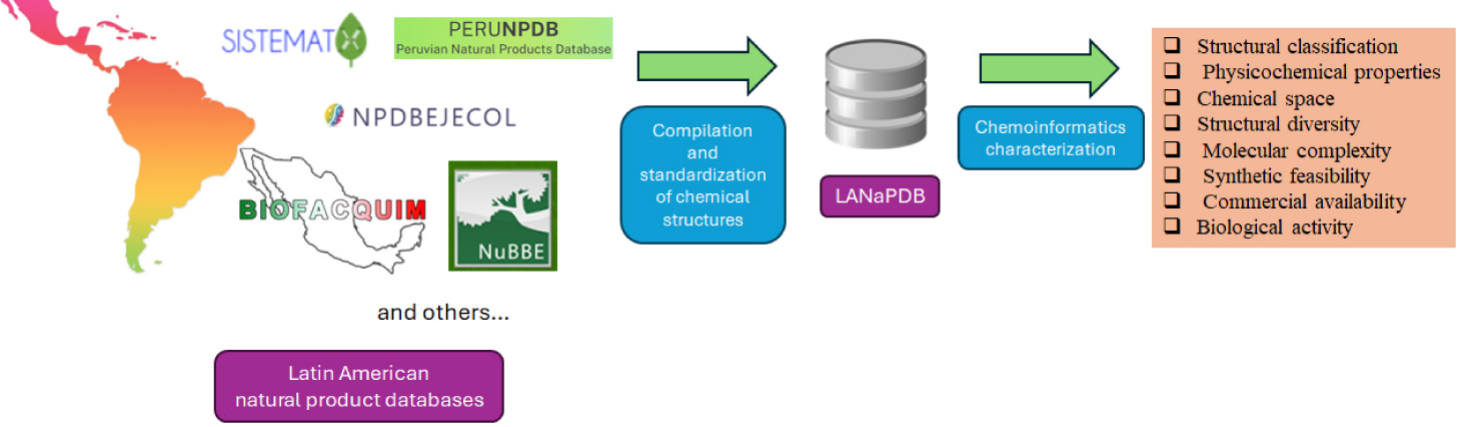

Natural product (NP) databases are crucial tools in computer-aided drug design (CADD). Over the past decade, there has been a worldwide effort to assemble information regarding natural products (NPs) isolated and characterized in certain geographical regions. In 2023, it was published LANaPDB, and to our knowledge, this is the first attempt to gather and standardize all the NP databases of Latin America. Herein, we present and analyze in detail the contents of an updated version of LANaPDB, which includes 619 newly added compounds from Colombia, Costa Rica, and Mexico. The present version of LANaPDB has a total of 13 578 compounds, coming from ten databases of seven Latin American countries. A chemoinformatic characterization of LANaPDB was carried out, which includes the structural classification of the compounds, calculation of six physicochemical properties of pharmaceutical interest, and visualization of the chemical space by employing and comparing two different fingerprints (MACCS keys (166-bit) and Morgan2 (2048-bit)). Furthermore, additional analyses were made, and valuable information not included in the first version of LANaPDB was added, which includes structural diversity, molecular complexity, synthetic feasibility, commercial availability, and reported and predicted biological activity. In addition, the LANaPDB compounds were cross-referenced to two of the largest public chemical compound databases annotated with biological activity: ChEMBL and PubChem.

## Introduction

Historically, natural products (NPs) have been the largest source of inspiration for the design of new drugs. In recent years (2018 compared to 2006), there has been a significant increase in the number of NP-based drugs.^1^ The recent technological advances, especially in the artificial intelligence (AI)^2^ and chemoinformatics^3^ areas, have boosted the NP-based computer-aided drug design (CADD). Among the recent progress in AI, the development of machine learning models to predict the target proteins of natural products stands out.^4^ During the severe acute respiratory syndrome coronavirus 2 (SARS-CoV-2) pandemic outbreak, the NP-based CAAD represented a main approach in the design and identification of lead compounds against the virus.^5−8^ Natural product (NP) databases are crucial tools for CADD since they provide access to thousands of molecules. In the past few years, the number of NP databases has grown, and some of the databases already established are continuously being updated. Among the largest freely available NP databases is Supernatural 3.0, with 449 048 NPs.^9^ The collection of open natural products (COCONUT 1.0)^9^ contains 411 000 NPs, and the universal natural product database^10^ has 229 000 NPs. The universal natural product database is still accessible on another online repository.^11^ The NP activity and species source (NPASS) database^12^ has 94 413 NPs, of which 43 285 are annotated with biological activity information. The Hippo(crates)^13^ database contains 45 300 NPs, NP derivatives, and synthetic compounds, many of which are annotated with their biological targets. There are NP databases that contain NPs isolated and characterized in certain geographical areas. TCM@Taiwan^14^ is the largest database of NPs from China, many of them employed in traditional Chinese medicine and contains 58 000 compounds. IMPPAT 2.0^15^ is the largest compilation of NPs from India with 17 967 phytochemicals, employed in traditional Indian medicine. The largest collection of NPs from Africa is NANPDB,^16^ containing over 4500 molecules from North Africa; nonetheless, there are other minor African NP databases.^17−20^

Latin America is a region with extraordinary biodiversity and richness in endemic species. It is a region that may be home to at least a third of global biodiversity.^21^ Brazil, for example, is considered to host the earth’s richest flora, with at least 50 000 species or one-sixth of the planetary total. Another example is Ecuador, with its megadiverse flora comprising more than 25 000 plant species (and thus twice the number of plant species found in Europe). Ecuador also has the highest vertebrate species density worldwide.^22^ Therefore, Latin America is a major source of bioactive compounds. Moreover, it has been reported that several databases contain NPs isolated and characterized in Latin American countries. More than 92 molecules with therapeutic effects have been identified from Latin American NP databases.^23^ Just recently, an NP database from Argentina^24^ and Colombia^25^ was published. In 2023, the first version of LANaPDB was published, a compendium that aims to gather and standardize the NP databases of Latin America^26^ which was already included in COCONUT (https://coconut.naturalproducts.net/search?type=tags&q=Latin+America+dataset&tagType=dataSource).^9^ In early 2024, an update was reported regarding the NP-likeness profile of the database.^27^

Herein, we report a major update of LANaPDB,^23^ a compound collection that aims to gather and standardize all the Latin American NP databases. The analysis of the database includes the structural classification of the compounds, calculation of six physicochemical properties of pharmaceutical interest, and visualization of the chemical space by employing and comparing two different fingerprints (MACCS keys (166-bit) and Morgan2 (2048-bit)). Furthermore, additional analyses were made, and valuable information not included in the first version of LANaPDB was added, which includes structural diversity, molecular complexity, synthetic feasibility, commercial availability, and reported and predicted biological activity. Moreover, the database was cross-referenced to two of the largest public chemical compound databases annotated with biological activity: ChEMBL^28^ and PubChem.^29^

## Methods

The version of the Python programming language that was used for all of the analyses in this article is 3.10.7. The versions of the Python packages are RDKit (2022.03.5),^30^ MolVS (0.1.1),^31^ Venn (0.1.3),^32^ Plotly Express (0.4.1),^33^ Scikit-learn (1.2.2),^34^ NumPy (1.23.2),^35^ and Seaborn (0.12.2).^36^

### Database Update and Data Curation

The first version of LANaPDB had 12 959 NPs coming from nine different databases of six different Latin American countries.^23^ To the first version of LANaPDB, a new database was added: NPDB EjeCol, which is a compilation of NPs isolated and characterized in Colombia, specifically from the region known as the Coffee Region.^25^ This database is set to be published in 2024 and is accessible through an open-data portal (www.npdbejecol.com). Furthermore, the LANaPDB was updated with new NPs from Costa Rica (NAPRORE-CR) and Mexico (BIOFACQUIM). In total, 619 new compounds were added to LANaPDB, resulting in a total of 13 578 NPs in the second version of the database. The curation of the second version of LANaPDB was carried out with the same workflow employed in the first version of the database.^26^ The process was performed in the Python programming language, employing the RDKit and MolVS packages. The standard curation process of MolVS was implemented through the standardize_smiles function included in this Python package, which includes and implements some functions from RDKit (SanitizeMol, RemoveHs, and AssignStereochemistry) and MolVS (disconnect, normalize, and reionize): verify and correct valencies, aromaticity, and hybridization, removal of explicit hydrogens, disconnection of covalent bonds between metals and organic atoms (the disconnected metal is removed later), application of normalization rules (transformations to correct common drawing errors and standardization of functional groups), reionization (ensure the strongest acid groups protonate first in partially ionized molecules), and recalculation of the stereochemistry (ensures the preservation of the original stereochemistry). From the molecules that are fragmented, i.e., the molecules that used to be connected with metals or other salts, only the largest fragment is kept (choose function from MolVS) and an attempt is made to neutralize all the molecules of the database (uncharge function from MolVS). The canonical SMILES strings were retrieved (MolToSmiles function of RDKit), and these are the SMILES strings included for every LANaPDB compound. The canonical tautomer was determined (canonicalize function from MolVS), and from the InChIKey strings of the canonical tautomer, the duplicate compounds were removed. The canonical tautomers were used only as part of the duplicate compound removal process; thus, the reported structures of the LANaPDB compounds correspond to the canonical SMILES strings retrieved before the elimination of the repeated molecules and not to the structure of the canonical tautomer. The same curation workflow was applied to two reference data sets employed to compare LANaPDB: COCONUT 1.0^9^ and FDA-approved small-molecule drugs, version 5.1.10 (released by DrugBank in January 2023).^37^

**Figure 1:**
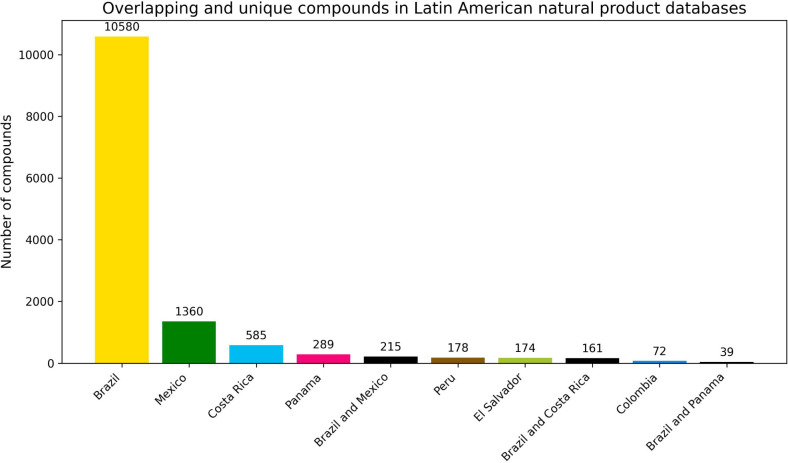
Bar chart showing the countries with the highest number of unique and overlapping compounds from the Latin American natural product databases contained in LANaPDB. The compounds are grouped according to the country of origin of the database.

To determine the number of unique and overlapping molecules in the different Latin American countries, the databases that encompass this version of LANaPDB were subjected to the above-described curation process; nonetheless, the duplicate removal step was omitted. Finally, from the molecule structures in the Python programming language employing the Venn package, the unique and overlapping molecules were determined (Figure 1).

### Structural Classification

The freely available online server NPClassifier^38^ was employed to perform the structural classification of the LANaPDB compounds. NPClassifier is a deep neural network-based structural classification tool for NPs. The distribution of the classified compounds was represented with pie plots constructed in Python using the Plotly Express package.

### Physicochemical Properties

The following physicochemical properties of pharmaceutical interest were calculated in Python employing the RDKit package: SlogP,^39^ molecular weight (MW), topological polar surface area (TPSA),^40^ rotatable bonds (Rb), hydrogen bond acceptors (HBA), and hydrogen bond donors (HBD). The distribution of the physicochemical properties was depicted with violin plots,^41^ constructed in the Python programming language with the Scikit-learn package.

### Chemical Space Visualization

The visualization of the chemical space of LANaPDB was made using the TMAP (Tree MAP) algorithm^42^ from the MACCS keys^43^ and Morgan2^44^ fingerprints. The determination of both fingerprints was made using the Python programming language with the RDKit package. The construction of the TMAP was made with Python, following the reported protocol.^42^ The results were compared with two reference data sets: COCONUT^9^ and FDA-approved small-molecule drugs, version 5.1.10 (released by DrugBank in January 2023).^37^

### Cross-References to Other Databases

The cross-references to PubChem^29^ and ChEMBL^28^ identification (ID) codes were requested and retrieved from the respective websites of both databases. The request and retrieval of the ID codes were made in the Python programming language, employing the corresponding application programming interface (API) for PubChem and ChEMBL. The InChIKey strings of the LANaPDB compounds were utilized to make the requests with the PubChem and ChEMBL application programming interfaces (APIs). The InChIKey strings were calculated in the Python programming language, employing the RDKit package.

### Commercial Availability and Chirality

The commercial availability of every compound of LANaPDB was obtained from the PubChem website.^29^ It is not information that can be retrieved with the PubChem API. Therefore, the Python programming language was used to retrieve the commercial availability, but without using the PubChem API. The classification of every compound based on chirality was made in Python, employing the function Chem.FindMolChiralCenters of the RDKit package.

### Biological Activity

The biological activity of the LANaPDB compounds was retrieved from ChEMBL, version 34, employing two different approaches. In the first approach, with the Python programming language, employing the ChEMBL API, from the InChIKey strings the reported biological activity of the LANaPDB compounds was requested and retrieved from the ChEMBL database website. In the second approach, in the Python programming language employing the RDKit package, it was determined if the SMILES strings of the LANaPDB molecules contained the SMILES strings of the ChEMBL bioactive rings reported by Ertl.^45^

### Structural Diversity

The Bemis and Murcko scaffolds^46^ were determined from the SMILES strings in the Python programming language with the RDKit package. The area under the curve (AUC) was obtained from the cumulative scaffold recovery (CSR) curves with the trapezoidal rule in the Python programming language with the trapz function of the numpy package. The fraction of scaffolds to retrieve 50% of the compounds in the database (*F*_50_) metric was obtained from the CSR curves by interpolating the *x*-axis value of 0.5 to find the corresponding *y*-axis value, in the Python programming language with the interp function of the numpy package. The MACCS keys (166-bit) fingerprint and the paired Tanimoto similarity^47^ were calculated in the Python programming language with the RDKit package. The paired Tanimoto similarity calculation for the COCONUT 1.0 data set was made with a random sample of 10% (with more than 40 000 compounds) that represents the diversity of the whole database.^48^

### Molecular Complexity and Synthetic Feasibility

The normalized spacial score (nSPS)^49^ and synthetic accessibility score (SAscore)^50^ were determined in the Python programming language with the RDKit package, employing the SpacialScore and sascorer^51^ functions. The kernel density estimate (KDE) plots^52^ were constructed in the Python programming language with the Seaborn package.

## Results and Discussion

### Database Update and Data Curation

The first version of LANaPDB comprised 12 959 compounds.^26^ This reported update includes 619 new compounds, resulting in a total of 13 578 compounds in its second version published in early 2024.^27^ A new data set was included: NPDB EjeCol, which contains NPs from foods and plants isolated and characterized in Colombia, from the Coffee Region (Eje Cafetero). Moreover, the database was updated with new NPs from Costa Rica (NAPRORE-CR) and Mexico (BIOFACQUIM). Table 1 shows the ten Latin American NP databases currently contained in LANaPDB. Initially, 1707 compounds were considered for the update of LANaPDB from the two updated databases, BIOFACQUIM and NAPRORE-CR, and the new database NPDB EjeCol. Nevertheless, from the initial 1707 compounds, 1088 molecules were duplicates and were no longer included. The remaining 619 molecules were added to LANaPDB.

**Table 1:** Natural Product Databases in the Updated Version of LANaPDB

Database	Number of compounds	Source	General description	References
NuBBE_DB_ (Brazil)	2223	plants, microorganisms, terrestrial and marine animals	Natural products of Brazilian biodiversity. Developed by the São Paulo State University and the University of São Paulo.	(53,54)
SistematX (Brazil)	9514	plants	Database composed of secondary metabolites and developed at the Federal University of Paraiba.	(55,56)
UEFS (Brazil)	503	plants	Natural products that have been separately published, but there is no common publication or public database for it. Developed at the State University of Feira de Santana.	(57)
NPDB EjeCol (Colombia)	236	plants, plants-derived food	Natural products and foods derived from plants present in the Eje Cafetero Región of Colombia, database created and curated at the Technological University of Pereira.	(25)
NAPRORE-CR (Costa Rica)	∼1600	plants, microorganisms	Developed in the CBio3 and LaToxCIA Laboratories of the University of Costa Rica.	a
LAIPNUDELSAV (El Salvador)	214	plants	Developed by the Research Laboratory in Natural Products of the University of El Salvador.	a
UNIIQUIM (Mexico)	1112	plants	Natural products isolated and characterized at the Institute of Chemistry of the National Autonomous University of Mexico.	(58)
BIOFACQUIM (Mexico)	750	plants, fungus *Propolis*, marine animals	Natural products isolated and characterized in Mexico at the School of Chemistry of the National Autonomous University of Mexico and other Mexican institutions.	(59,60)
CIFPMA (Panama)	363	plants	Natural products that have been tested in over 25 in vitro and in vivo bioassays for different therapeutic targets, developed at the University of Panama.	(61,62)
PeruNPDB (Peru)	280	animals, plants	Natural products representative of Peruvian biodiversity. Created and curated at the Catholic University of Santa Maria.	(63)

a The database has not been published yet.

The number of unique and overlapping molecules in every Latin American country was determined from the databases that contain LANaPDB, and Figure 1 shows the countries with the highest number of unique and overlapping compounds. It was found that the number of unique molecules is associated with the number of molecules in the country. Brazil is the country with the most unique molecules (10 580), followed by Mexico (1360), Costa Rica (585), Panama (289), Peru (178), El Salvador (174), and Colombia (72). Furthermore, it was found that Brazil has the highest number of overlapping compounds with other countries (Mexico: 215, Costa Rica: 161, and Panama: 39), which can be attributed to the fact that Mexico, Costa Rica, and Panama have the largest number of reported compounds after Brazil (Table 1). Nonetheless, it can also imply that Brazil shares flora and fauna with these three countries, with Mexico being the country with the highest number of shared compounds. There is a very small number of overlapping compounds among the other countries, with almost zero overlapping compounds in most cases. A possible explanation is that the remaining countries (Colombia, El Salvador, Panama, and Peru) have much fewer reported compounds than Brazil, Costa Rica, and Mexico.

### Structural Classification

**Figure 2:**
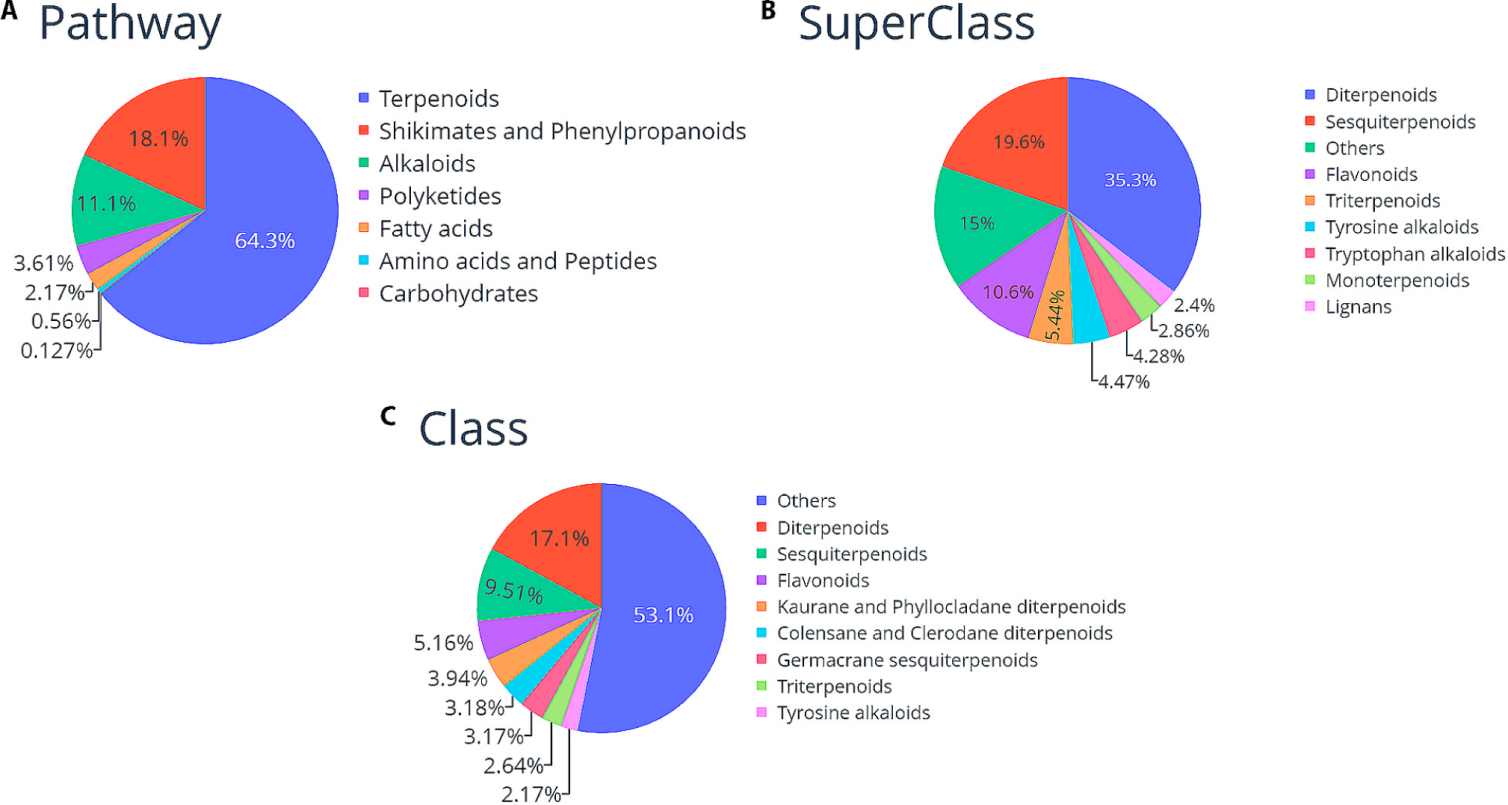
Pie charts showcasing the distribution of the LANaPDB compounds, according to a classification system^38^ based on the literature from the specialized metabolism of the producing organisms. **A**) Pathway: related to the nature of the biosynthetic pathway. **B**) SuperClass: associated with chemical properties or chemotaxonomic information, and **C**) Class: correlated to structural details.

The compounds in LANaPDB were structurally classified according to a classification system based on the literature on the specialized metabolism of plants, marine organisms, fungi, and microorganisms. The classification system is divided into three hierarchical levels: pathway (nature of the biosynthetic pathway), superclass (chemical properties or chemotaxonomic information), and class (structural details). At the three hierarchical levels, the predominant compounds are terpenoids (Figure 2). At the hierarchical level of the pathway, terpenoids, shikimates, phenylpropanoids, and alkaloids encompass more than 90% of the total compounds. At the hierarchical level of superclass and class, terpenoids and flavonoids were the predominant compounds (Figure 2). The above was expected because terpenoids are the predominant secondary metabolites produced by natural sources.^64^ Compared to the previous version of LANaPDB, the above tendencies have not changed.^26^

### Physicochemical Properties

**Figure 3:**
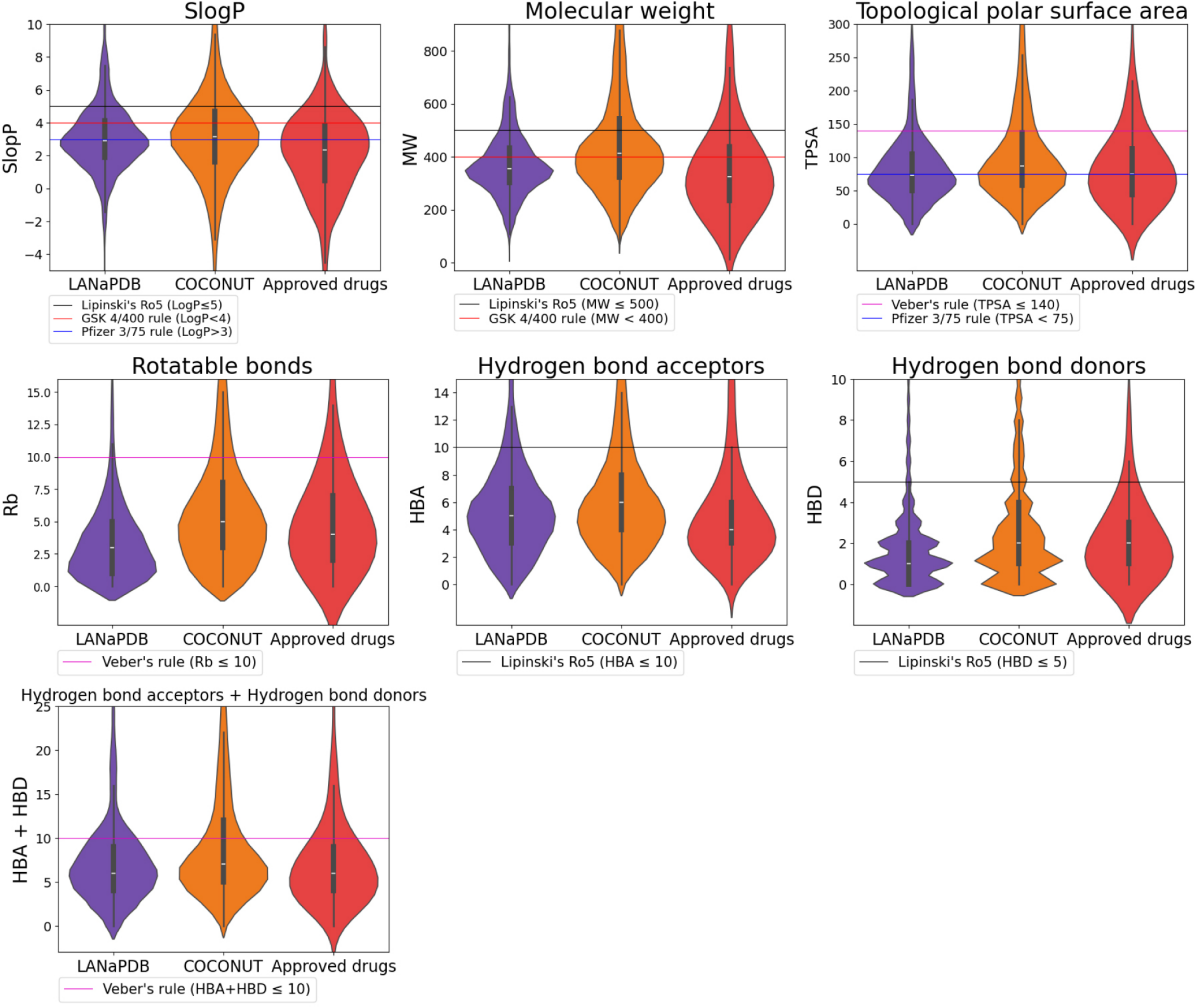
Violin plots summarizing the distribution of seven physicochemical properties of pharmaceutical interest in the compounds from three databases: LANaPDB, COCONUT, and FDA-approved small-molecule drugs.

**Figure 4:**
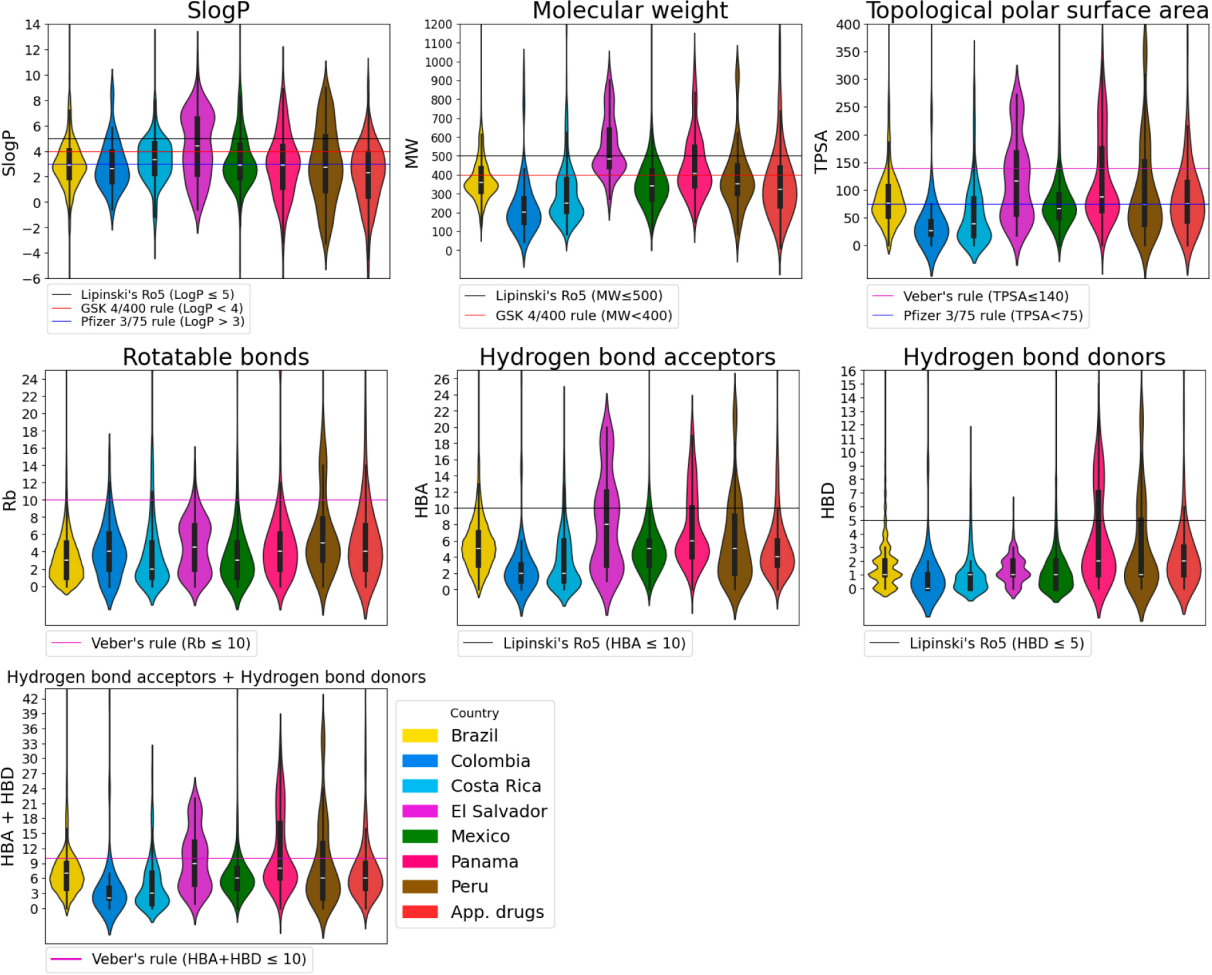
Violin plots summarizing the distribution of seven physicochemical properties of pharmaceutical interest of the compounds in LANaPDB and FDA-approved small-molecule drugs (App. drugs). The databases that encompass LANaPDB for every country: Brazil (NuBBEDB, SistematX, and UEFS), Colombia (NPDB EjeCol), Costa Rica (NAPRORE-CR), El Salvador (LAIPNUDELSAV), Mexico (UNIIQUIM and BIOFACQUIM), Panama (CIFPMA), and Peru (PeruNPDB).

We calculated the physicochemical properties of pharmaceutical interest for the LANaPDB compounds and compared them with two reference data sets: COCONUT^9^ and FDA-approved small-molecule drugs.^37^Figures 3 and 4 show the distribution of the calculated physicochemical properties: SlogP,^39^ molecular weight (MW), topological polar surface area (TPSA),^40^ number of rotatable bonds (Rb), hydrogen bond acceptors (HBA), and hydrogen bond donors (HBD). The violin plots (Figures 3 and 4) are marked with a horizontal line indicating the limits of some drug-likeness rules of thumb: Lipinski’s rule of 5 (Ro5),^65,66^ Veber’s rules,^67^ GlaxoSmithKline’s (GSK) 4/400 rule,^68^ and Pfizer 3/75 rule.^69^ Physicochemical properties within the limits of either Lipinski’s, Veber’s, or GSK rules are usually related to good oral bioavailability. The fulfillment of these rules of thumb is associated with the improvement of the following parameters: aqueous solubility and intestinal permeability (Lipinski’s Ro5); passive membrane permeation (Veber’s rules); absorption, distribution, metabolism, excretion, and toxicity (ADMET) profile (GlaxoSmithKline’s 4/400 rule); and toxicity (Pfizer 3/75 rule). In Figure 3, noticeable changes in the distribution of the physicochemical properties of LANaPDB are not appreciated compared to the previous version.^26^ This can be attributed to the fact that the terpenoids remain as the prevalent compounds (Figure 2). The physicochemical properties related to the Ro5 (SlogP, MW, HBA, and HBD), Veber’s rules (HBA + HBD, TPSA, and Rb), and the GlaxoSmithKline’s 4/400 rule (SlogP and MW) are within the limits of these three rules of thumb for most of the compounds in the three databases (Figure 3). Therefore, the aqueous solubility, intestinal permeability, oral bioavailability, and in general the ADMET profile are desirable for the three databases. Moreover, the three databases have a similar distribution for these physicochemical properties. Nevertheless, regarding the Pfizer 3/75 rule, which is related to toxicity, just approximately half of the compounds in the three databases satisfy the requirements of SlogP > 3 and TPSA < 75 (Figure 3). Therefore, according to the obtained values of SlogP and TPSA, considering the Pfizer 3/75 rule, half of the compounds in the three databases have a desirable toxicity profile. Regardless, half of the FDA-approved small-molecule drugs satisfy the Pfizer 3/75 rule; therefore, the compounds that do not satisfy this rule are still worth consideration in drug design because the toxicity is not just related to the SlogP and TPSA.

The LANaPDB compounds presented fewer rotatable bonds compared to COCONUT. This result can be attributed to the fact that the number of compounds in COCONUT is much larger compared to LANaPDB and as a consequence, the diversity in the structures contributes to a wider distribution in the rotatable bonds. Nonetheless, comparing this version of LANaPDB to the prior version of the database, the distribution of rotatable bonds is the same, which can be attributed to the fact that in both versions of the database, the terpenoid compounds are predominant and on average, they have fewer than four rotatable bonds. Regardless, the rotatable bonds in LANaPDB fulfill Veber’s rules (Rb < 10). Therefore, it is expected to have good passive membrane permeation.

In the case of the individual countries that encompass LANaPDB, a similar behavior was observed for the physicochemical properties, where most of the compounds satisfy the Ro5, Veber’s rules, and GlaxoSmithKline’s 4/400 rule, but a lower proportion satisfies the Pfizer 3/75 rule. Nevertheless, Panama, compared to the other countries, shows a higher proportion of compounds with higher SlogP and MW, which can be detrimental to intestinal permeability and, in general, to the ADMET profile; therefore, other routes of administration should be considered for these compounds, for instance, the nasal delivery route.^70^ Therefore, most of the LANaPDB compounds have a desirable physicochemical profile that allows them to be employed in the design of new drugs, either as potential drug candidates or as a starting point to design semisynthetic drugs or pseudo-NP.

Figure 4 shows the distribution of the physicochemical properties of pharmaceutical interest of LANaPDB, considering the seven countries individually. For comparison, the distribution of the compounds in the FDA-approved drugs is included. In general, it is observed that the distribution of compounds is mainly focused on regions that fulfill the drug-likeness rules of thumb. Nonetheless, El Salvador is a country with many compounds outside of the drug-likeness parameters considering the SlogP and MW. In the current version of LANaPDB, new compounds from Costa Rica and Mexico were added; nevertheless, the distribution of the physicochemical properties of the compounds of both countries compared to the previous version of LANaPDB^26^ remained without significant changes. The distribution of the physicochemical properties of the compounds of the new country added to the current version of LANaPDB, Colombia, is such that most of the compounds fulfill the drug-likeness rules of thumb.

### Chemical Space Visualization

**Figure 5:**
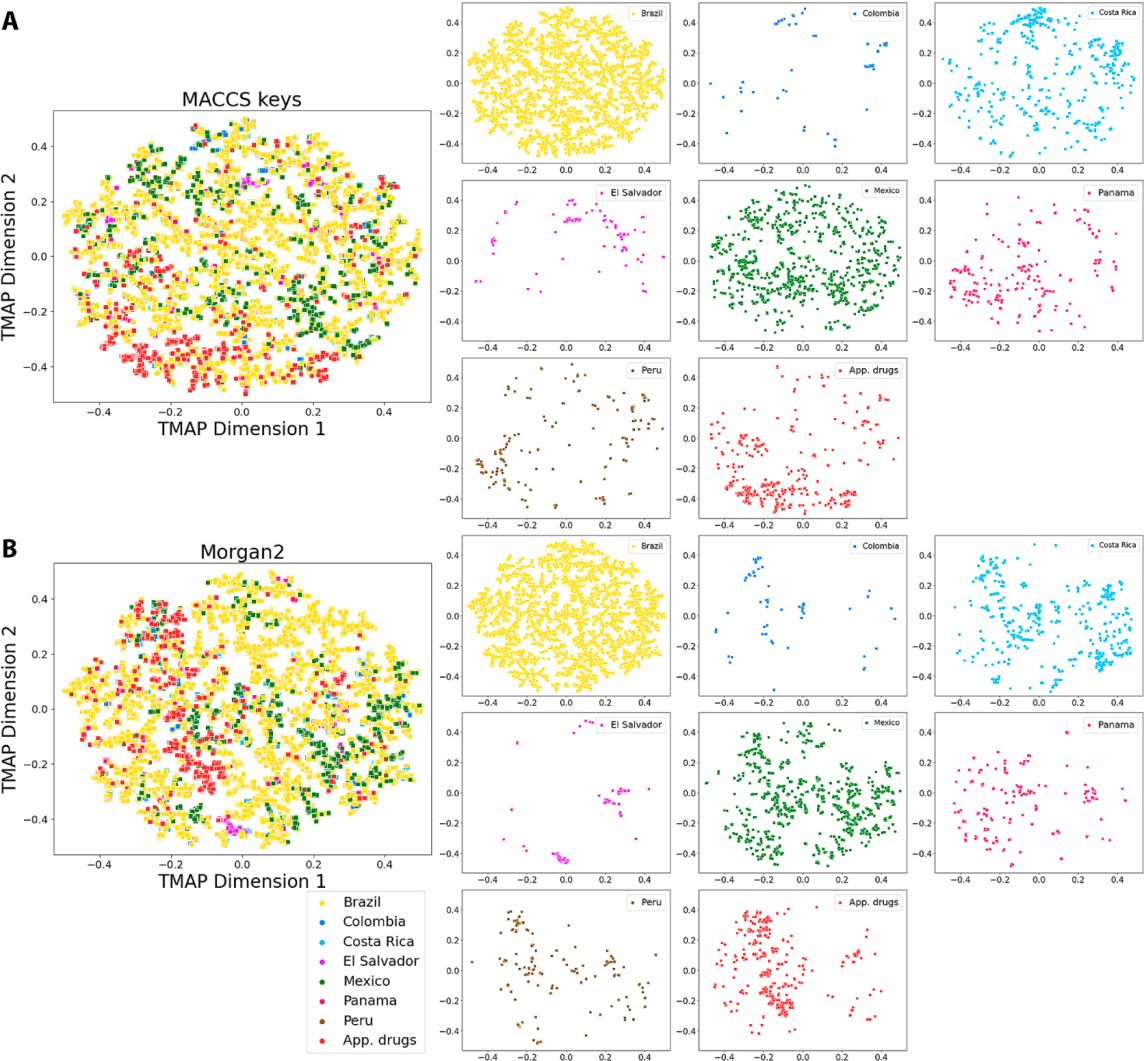
Tree MAP of LANaPDB and the comparison with FDA-approved small-molecule drugs, generated from **A**) MACCS keys (166-bit) and **B**) the Morgan2 (2048-bit) fingerprint. An interactive version of the TMAP is available for free download (MACCS keys (166-bit): https://github.com/alexgoga21/LANaPDB-version-2/blob/main/Interactive%20TMAP%20MACCS%20keys.html and Morgan2: https://github.com/alexgoga21/LANaPDB-version-2/blob/main/Interactive%20TMAP%20Morgan2.html) (to open the interactive map, download the file and open it in a web explorer; zoom in option is available with the mouse scroll).

**Figure 6:**
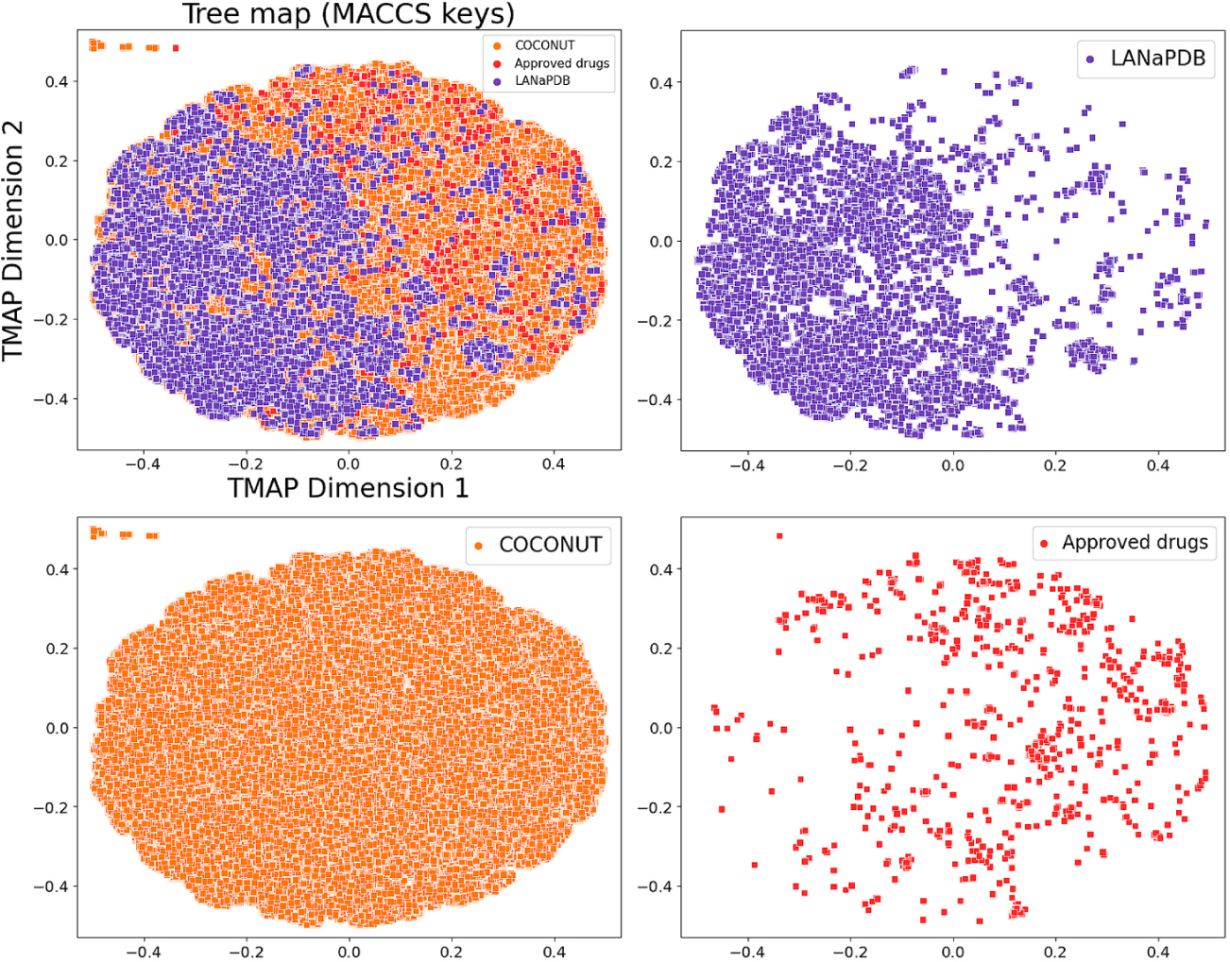
Tree MAP of LANaPDB and the comparison with COCONUT and FDA-approved small-molecule drugs, generated from the MACCS keys (166-bit) fingerprint.

Figure 5 shows the TMAP of LANaPDB generated from the MACCS keys (166-bit),^43^ Morgan2^44^ fingerprints, and their comparison with the FDA-approved small-molecule drugs.^37^ The structural features of the compounds are not necessarily correlated to the numerical values of the *x* and *y* axes. Therefore, an interactive version of the TMAP is also freely available for download (MACCS keys (166-bit): https://github.com/alexgoga21/LANaPDB-version-2/blob/main/Interactive%20TMAP%20MACCS%20keys.html and Morgan2: https://github.com/alexgoga21/LANaPDB-version-2/blob/main/Interactive%20TMAP%20Morgan2.html) (to open the interactive map, download the file and open it in a web explorer; zoom in option is available with the mouse scroll). MACCS keys (166-bit) were chosen for their capacity to capture structural features from well-known predefined fragments and Morgan2 (2048-bit) for their efficiency in capturing detailed structural features. In the interactive version of Figure 5, it can be appreciated that the TMAP effectively accomplished the clustering of structurally similar compounds in “branches” for both fingerprints. Therefore, both fingerprints showed similar and very good capacities to capture the structural features of NPs. Neither MACCS keys (166-bit) nor Morgan2 (2048-bit) fingerprints appear to outperform the other in capturing structural features according to both interactive plots of Figure 5. Figure 5 shows that all of the countries and the approved drugs with both fingerprints overlap with the Brazilian NPs. Therefore, Brazil is the country with the highest structural diversity of NPs according to the TMAP. Moreover, the compounds for each of the seven Latin American countries with both fingerprints are in general not focused on a certain region of the chemical space. Instead, they are distributed across the chemical space and, in many cases, clustered, forming branches of structurally similar compounds. Besides, all the Latin American countries partially overlap with the approved drugs in specific regions for both fingerprints. Figure 6 depicts the comparison of LANaPDB with COCONUT and the approved drugs with the MACCS keys (166-bit) fingerprint. LANaPDB totally overlaps with COCONUT. Interestingly, the overlap of LANaPDB with COCONUT is mostly in a well-defined area (left side of the TMAP), which shows that COCONUT covers a huge area (right side of the TMAP) of the chemical space not covered by LANaPDB. It is important to consider that COCONUT has more than 400 000 compounds and LANaPDB 13 578. In Figure 6, it is appreciated that the approved drugs are distributed across the chemical space, overlapping LANaPDB and COCONUT in different regions.

### Cross-References to Other Databases

The LANaPDB compounds were cross-referenced to two of the biggest publicly available chemical compound databases annotated with biological activity: PubChem, version 2024^29^ and ChEMBL, version 34.^28^ From both databases, the ID code was retrieved. The ID code allowed to identify and differentiate every single compound. In the case of PubChem, the ID codes are known as CID (compound identification) and SID (substance identification). From all the LANaPDB compounds, 71.71% of the ID codes were successfully retrieved from PubChem and 23.69% from ChEMBL.

Therefore, most of the LANaPDB compounds can be found in PubChem, and just a minority in ChEMBL. To consult additional information for the LANaPDB compounds in PubChem and ChEMBL, it is just needed to type the corresponding ID code in the respective websites of both databases. The SMILES strings contained in ChEMBL and the ones determined for the compounds of LANaPDB versions 1 and 2 were obtained with RDKit, which uses its own canonicalization method; thus, they are comparable to each other. The additional information that can be checked in PubChem for the LANaPDB compounds includes spectral information, toxicity, and patents. ChEMBL contains information about metabolism, target predictions, drug indications, and mechanism of action.

### Commercial Availability and Chirality

It was found that 70.5% of the LANaPDB compounds are commercially available, as annotated on the PubChem website. The information about the companies that sell the individual molecules can be consulted on the PubChem website, from the PubChem ID codes added to LANaPDB. Moreover, all the molecules were classified into three categories: achiral (16.16%) and chiral with chirality annotated (55.53%) or not annotated (28.31%).

### Biological Activity

The biological activity of the LANaPDB compounds was retrieved from ChEMBL, with two different approaches. In the first one, the biological activity was retrieved from the ChEMBL website with the ChEMBL API. It was found that only 0.29% of the LANaPDB compounds (39 molecules) have a reported biological activity that can be retrieved with the ChEMBL API. These compounds have up to three biological activities reported. The most common biological activities are pharmaceutical aid (flavor) (4 compounds), pharmaceutical aid (solvent) (3 compounds), antifungal (3 compounds), pharmaceutical aid (antimicrobial agent) (2 compounds), pharmaceutical aid (emulsion adjunct) (2 compounds), and inhibitor (alpha-glucosidase) (2 compounds).

**Chart 1:**
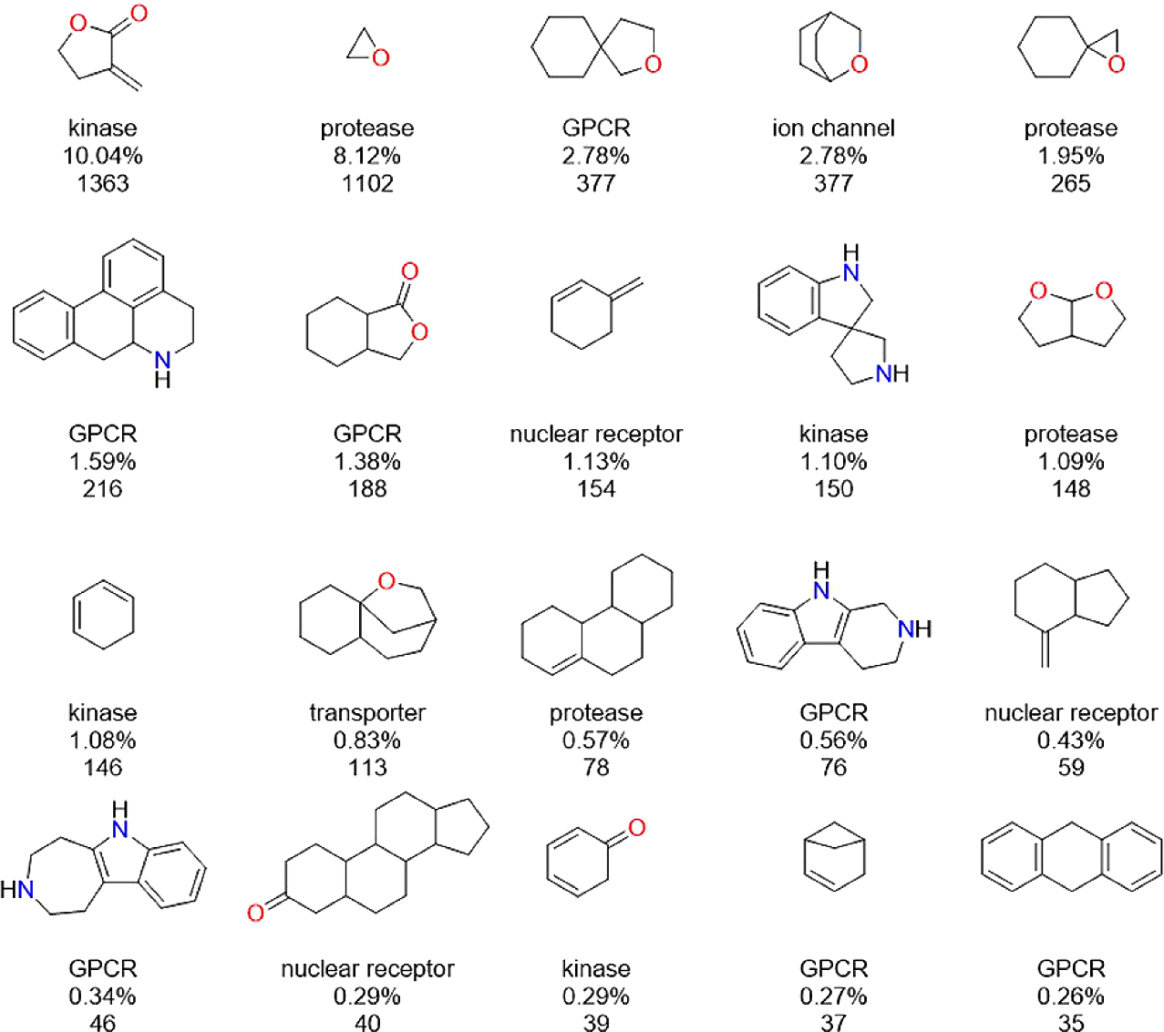
20 Most Abundant Bioactive Ring Systems (with Reported Biological Activity in ChEMBL) in LANaPDB, their Biological Targets, Percentage of Occurrence, and the Total Number of Compounds that Contain the Ring System in LANaPDB

**Figure 7:**
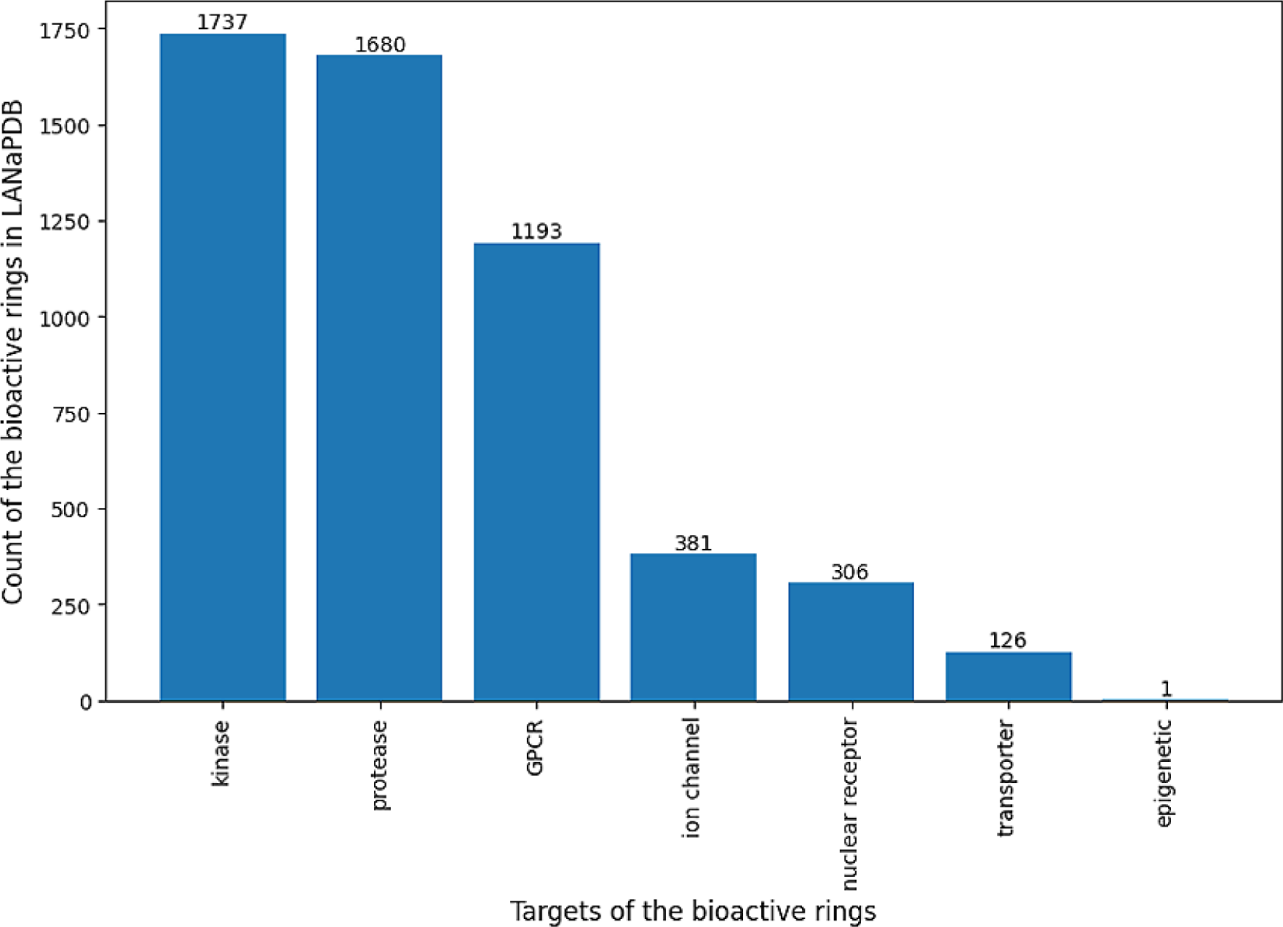
Histogram that shows the occurrence of bioactive rings (with reported bioactivity in ChEMBL) in LANaPDB and their biological target. Consider that every molecule can have more than one bioactive ring in its structure.

The second approach was based on a study by Peter Ertl who previously extracted the ring systems from the molecules in ChEMBL (version not specified) and associated them with their reported bioactivity in ChEMBL against the following biological target families: G protein-coupled receptor (GPCR), kinase, protease, nuclear receptor, ion channel, transporters, and epigenetic targets.^45^ For LANaPDB, it was determined which compounds contain the bioactive ring systems reported by Ertl. It was found that 31.51% of the LANaPDB compounds (4279 molecules) have bioactive ring systems. Chart 1 shows the 20 most abundant ring systems found in the LANaPDB compounds; the most abundant ring system agrees with the most abundant pathway found in LANaPDB (Figure 2) as it pertains to bioactive sesquiterpenic lactones.^71^Figure 7 shows that the bioactive rings in LANaPDB mainly target kinases, proteases, and G protein-coupled receptors (GPCRs), which is related to the fact that they are among the most extensively studied drug targets. GPCRs are the most studied drug targets,^72^ and approximately up to 2018, 35% of the approved drugs (∼700) target GPCRs.^73^ Kinases are the second most therapeutically targeted group of proteins, after GPCRs, and up to 2023, 98 kinase inhibitors were approved.^74^ Proteases are another extensively studied therapeutic target; up to 2011, 12 drugs that target proteases had been approved.^75^

It is important to take into account that the remaining percentage of compounds (68.49%) without bioactive ring systems are not necessarily inactive compounds; they may be active but against other biological targets different from the ones that were reported by Ertl.^45^ Take into account that the currently known scaffold space is far from being fully explored. This is exemplified by the fact that in 2024, Ertl published a database of four million medicinal chemistry-relevant scaffolds that are not included in ChEMBL and PubChem.^76^

### Structural Diversity

The structural diversity of LANaPDB was quantified with two types of molecular representations: molecular scaffolds and fingerprints. The diversity was compared to those of COCONUT and FDA-approved small-molecule drugs. The scaffold diversity of all data sets was measured with CSR curves that represent the fraction of molecules in the data set contained in a fraction of scaffolds. To generate the CSR curves, the scaffolds are ordered by their frequency of occurrence (most to least common). Then, the fraction of scaffolds is plotted on the *x*-axis, and the fraction of compounds that contain those scaffolds is plotted on the *y*-axis. Two metrics were obtained from the CSR curves: AUC and *F*_50_ (i.e., if a data set has *F*_50_ = 0.43, 50% of the compounds in the data set are distributed in 43% of the scaffolds). A data set with maximum diversity would contain a different scaffold for each molecule in the library, and the curve would be a diagonal with an AUC of 0.5. As the scaffold diversity decreases, the curve will move away from the diagonal. The minimum diversity would be a data set in which all of the compounds have the same scaffold. In this case, the CSR function would be a vertical line with an AUC equal to 1.0. The fingerprint-based diversity was assessed with the mean of the paired Tanimoto similarity (MPTS), using the MACCS keys (166-bit) fingerprint (mainly quantifies the side chain structural diversity).^77,78^

**Figure 8:**
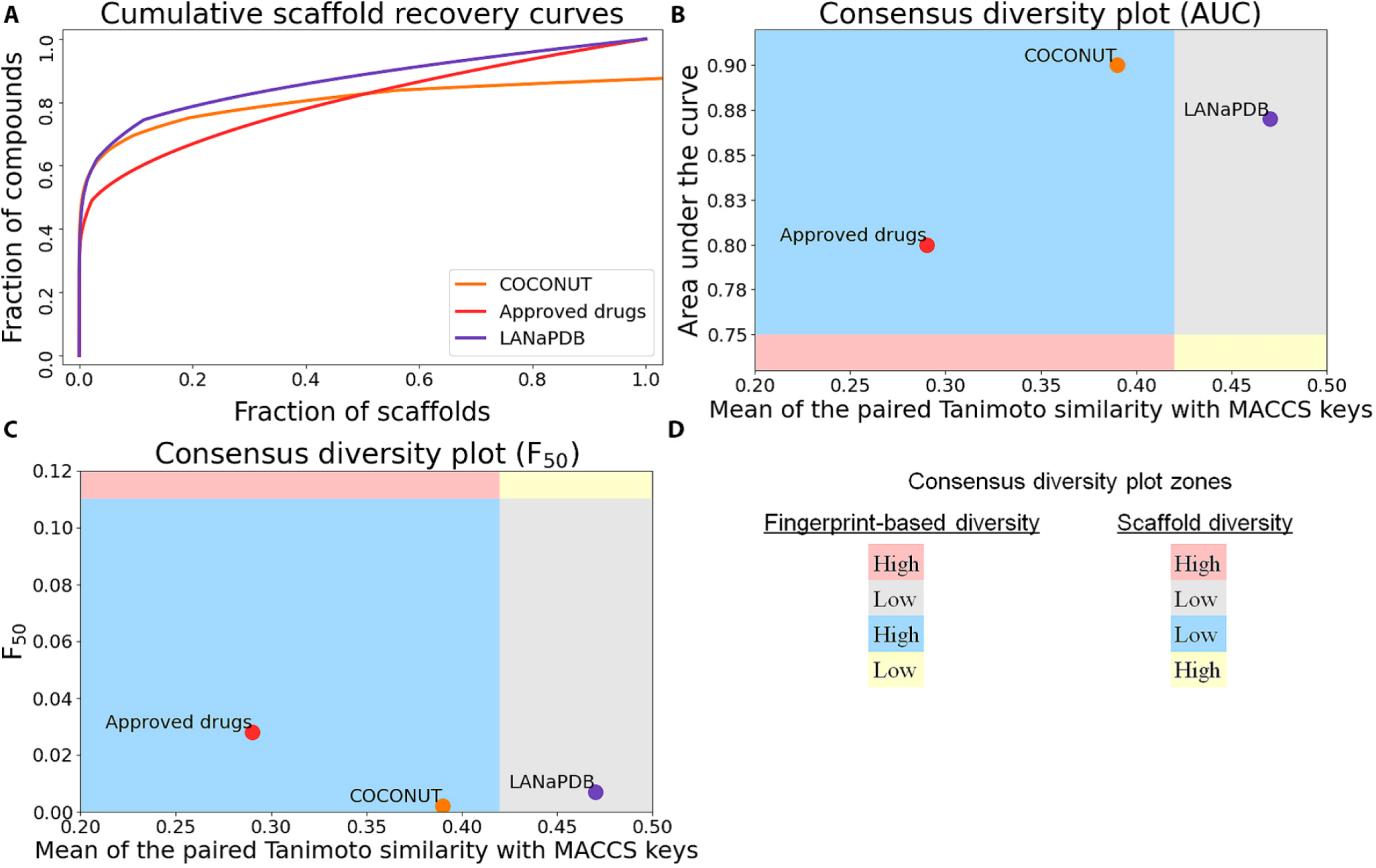
**A**) Cumulative scaffold recovery (CSR) curves of LANaPDB, COCONUT, and FDA-approved small-molecule drugs. Consensus diversity plots of LANaPDB, COCONUT, and FDA-approved small-molecule drugs, which describe the data set diversity considering the MACCS keys (166-bit) fingerprint, **B**) area under the curve, and **C**) the fraction of scaffolds to retrieve 50% of the database (*F*_50_). **D**) Degree of scaffold and fingerprint-based diversity in the consensus diversity plots’ quadrants.

In the consensus diversity plots (Figures 8B,C) it is shown that FDA-approved small-molecule drugs is the data set with the highest scaffold and fingerprint-based diversity (AUC = 0.80, *F*_50_ = 0.028, and MPTS = 0.29), followed by LANaPDB (AUC = 0.87, *F*_50_ = 0.007, and MPTS = 0.47) and COCONUT (AUC = 0.90, *F*_50_ = 0.002, and MPTS = 0.39). This result can be attributed to the fact that this data set contains not just NPs; instead, a significant proportion are NP derivatives and purely synthetic molecules,^79^ which increases the structural diversity. According to the MPTS metric, the side chain structural diversity of LANaPDB is lower than that of COCONUT. Nonetheless, considering the AUC and *F*_50_ metrics, LANaPDB has higher scaffold diversity than that of COCONUT; nevertheless, the difference between both databases considering these two metrics is small (ΔAUC = 0.03 and Δ*F*_50_ = 0.005). Therefore, the structural diversity of LANaPDB is very similar to COCONUT, with less side chain diversity and a little more scaffold diversity.

### Molecular Complexity and Synthetic Feasibility

**Figure 9:**
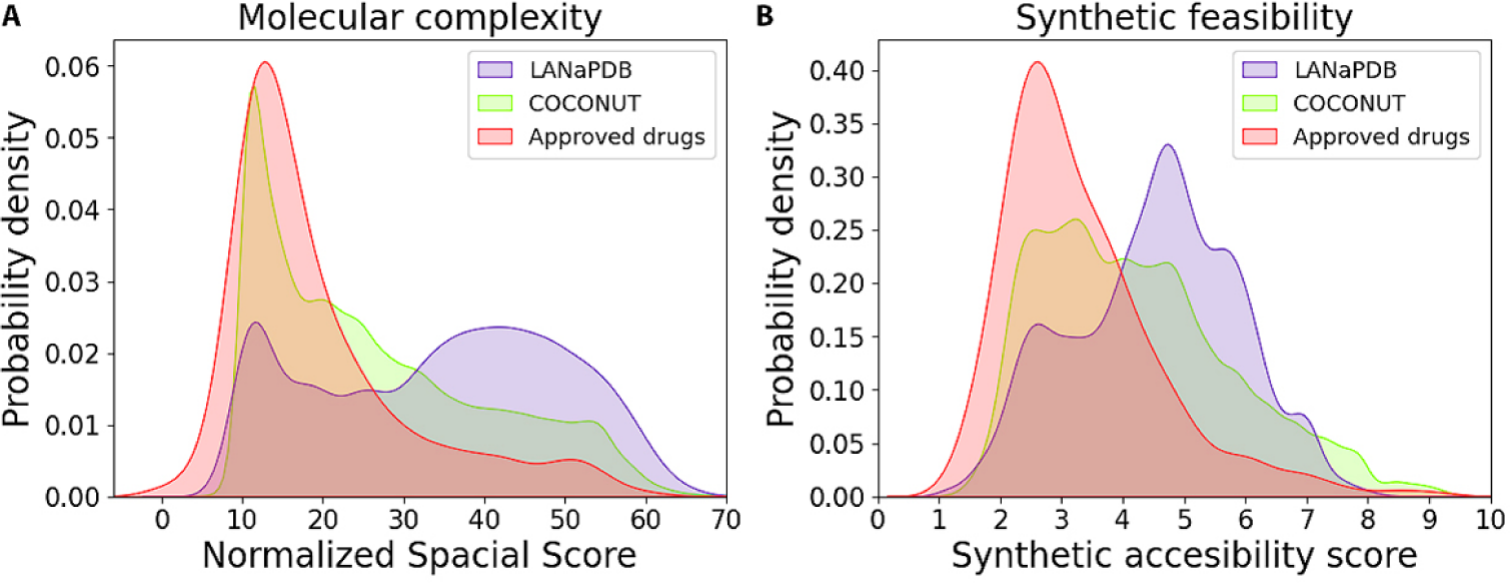
Kernel density estimate plots that represent the distribution of the **A**) normalized spacial score and **B**) synthetic accessibility score of LANaPDB, COCONUT, and FDA-approved small-molecule drugs.

Molecular complexity can be quantified using different metrics.^80^ In this work, as a quantitative measure of molecular complexity, we employed the recently developed metric nSPS.^49^ The synthetic feasibility was determined by calculating the SAscore.^50^ The distribution of both metrics was represented with KDE plots, which represent the data using continuous probability density curves (Figure 9). nSPS takes into account the atom hybridization, stereoisomerism, presence and complexity of aromatic or nonaromatic rings, and the number of heavy-atom neighbors.^49^ As a reference, in an earlier study, it was found that the nSPS values of most of the approved drugs are between 10 and 20, and this has remained without any significant changes in the last eight decades.^81^ The nSPS values for the compounds of the three databases studied in this work are centered around 10 and 20 (Figure 9A). Thus, LANaPDB has a significant proportion of compounds with nSPS values between 10 and 20 (39.78%), and those compounds are expected to have a similar pharmacokinetic profile to the approved drugs according to the molecular similarity principle.^81^ Moreover, unlike the other two reference databases, the LANaPDB compounds presented mainly nSPS values around 30 and 50 (26.88%) (Figure 9A). Previously, it has been found that the ligand potency and target selectivity are maximized in compounds with nSPS values between 20 and 40.^49^ Therefore, LANaPDB has a significant proportion of compounds with nSPS values between 20 and 40 (37.95%), which are expected to have good potency and target selectivity. The nSPS value for each compound in LANaPDB is indicated in the publicly available database.

The synthetic feasibility was estimated with the SAscore, which considers the complexity of the molecular fragments, stereocomplexity, and molecule size. The synthetic feasibility is positively correlated with the SAscore, i.e., highest SAscores are associated with higher synthetic feasibility.^50^ In this work, approved drugs and COCONUT presented mainly SAscores between two and three (Figure 9B). The accumulation of SAscores of approved drugs and COCONUT in the same zone can be attributed to the fact that a large proportion of the approved drugs are NPs or NP-based molecules.^79^ The LANaPDB compounds have mostly SAscores around five, which implies that a significant proportion of the LANaPDB compounds have a synthetic feasibility higher than that of the approved drugs.

## Conclusions

LANaPDB was updated with 619 new molecules from Colombia, Costa Rica, and Mexico, resulting in a total of 13 578 compounds. It is highlighted that the addition of a new database of NPs from Colombia, NPDB EjeCol, is the first database that gathers NPs from Colombia. In the structural classification of the compounds, it was found that terpenoids are still the dominant compounds in the database. According to the calculated physicochemical properties of pharmaceutical interest, most of the LANaPDB compounds have a desirable physicochemical profile, which allows them to be employed in the design of new drugs. In the chemical space visualization, it was found that LANaPDB totally overlaps with COCONUT and partially overlaps with FDA-approved small-molecule drugs. Furthermore, MACCS keys (166-bit) and Morgan2 (2048-bit) showed similar and good capacities to capture structural features from the LANaPDB compounds. Moreover, the LANaPDB compounds were cross-referenced to ChEMBL and PubChem. It was found that 70.5% of the database molecules are commercially available, and the information regarding the vendors can be consulted on the PubChem website, employing the PubChem IDs that were added to the LANaPDB compounds. Only 39 molecules of LANaPDB have reported biological activity on ChEMBL; nonetheless, 4279 molecules have bioactive ring systems. From the structural diversity analysis, it was found that LANaPDB has less scaffold and fingerprint-based diversity than FDA-approved small-molecule drugs; nevertheless, compared to COCONUT, LANaPDB has less side chain diversity and a little more scaffold diversity. According to the molecular complexity of the molecules in the database, they are expected to have a similar pharmacokinetic profile to the approved drugs, and most of the compounds have high synthetic feasibility. LANaPDB is an ongoing project and is planned to keep updating with more compounds and adding more information, such as spectroscopic data and the ADMET profile.

## Data Availability

The LANaPDB database is publicly available at https://github.com/alexgoga21/LANaPDB-version-2. The whole database can be downloaded as an xlsx file at https://github.com/alexgoga21/LANaPDB-version-2/blob/main/LANaPDB%20version%202.xlsx. The interactive tree MAP can be downloaded as an html file at https://github.com/alexgoga21/LANaPDB-version-2/blob/main/Interactive%20TMAP%20MACCS%20keys.html and https://github.com/alexgoga21/LANaPDB-version-2/blob/main/Interactive%20TMAP%20Morgan2.html. To open the interactive map, download the file and open it in a web explorer; zoom in option is available with the mouse scroll. The first version of LANaPDB can be consulted on the COCONUT web server at https://coconut.naturalproducts.net/search?type=tags&q=Latin+America+dataset&tagType=dataSource.
